# A Feasibility Trial of Mental Health First Aid First Nations: Acceptability, Cultural Adaptation, and Preliminary Outcomes

**DOI:** 10.1002/ajcp.12241

**Published:** 2018-03-25

**Authors:** Claire V. Crooks, Andrea Lapp, Monique Auger, Kim van der Woerd, Angela Snowshoe, Billie Jo Rogers, Samantha Tsuruda, Cassidy Caron

**Affiliations:** ^1^ Western University London ON Canada; ^2^ Reciprocal Consulting Vancouver BC Canada; ^3^ University of Regina Regina SK Canada

**Keywords:** Indigenous peoples, Mental health literacy, Health promotion, Community, Mixed methods, Feasibility trial

## Abstract

The Mental Health First Aid First Nations course was adapted from Mental Health First Aid Basic to create a community‐based, culturally safe and relevant approach to promoting mental health literacy in First Nations contexts. Over 2.5 days, the course aims to build community capacity by teaching individuals to recognize and respond to mental health crises. This feasibility trial utilized mixed methods to evaluate the acceptability, cultural adaptation, and preliminary effectiveness of MHFAFN. Our approach was grounded in community‐based participatory research principles, emphasizing relationship‐driven procedures to collecting data and choice for how participants shared their voices. Data included participant interviews (*n* = 89), and surveys (*n* = 91) from 10 groups in four provinces. Surveys contained open‐ended questions, retrospective pre‐post ratings, and a scenario. We utilized data from nine facilitator interviews and 24 facilitator implementation surveys. The different lines of evidence converged to highlight strong acceptability, mixed reactions to the cultural adaptation, and gains in participants’ knowledge, mental health first aid skill application, awareness, and self‐efficacy, and reductions in stigma beliefs. Beyond promoting individual gains, the course served as a community‐wide prevention approach by situating mental health in a colonial context and highlighting local resources and cultural strengths for promoting mental well‐being.

## Highlights

- There are high rates of mental health challenges in many community and urban First Nations contexts.
- The lack of appropriate and effective services underscores the need for community‐based approaches.
- Mental Health First Aid First Nations adapted an existing program to include a cultural context.
- Some participants considered the cultural focus a strength, but a minority found it inadequate.
- Participants reported gains in knowledge, self‐efficacy and skills, and decreased stigma beliefs.

First Nations^1^ peoples in Canada experience a wide range of negative health outcomes at rates that are disproportionate to other Canadians (Adelson, 2005). The root of these health inequities is increasingly understood to lie within the impacts of colonization; this has included residential schools, the reserve system, cultural suppression, and deterioration of Indigenous healing practices, and contemporary and systemic forms of cultural discrimination. Data from the 2012 Aboriginal Peoples Survey indicated that familial residential school attendance was directly related to all five of the health outcomes examined, including self‐perceived health, mental health problems, psychological distress, suicidal ideations, and attempts (Hackett, Feeny, & Tompa, 2016). A recent scoping review of the effects of colonization on health and well‐being documented the negative impacts of residential schools on a range of physical and mental health outcomes (Wilk, Maltby, & Cooke, 2017). The 61 articles in this scoping review identified numerous negative impacts on emotional and mental well‐being, as indicated by rates of mental distress, depression, addictive behaviors and substance misuse, stress and suicidal behaviors. These negative impacts are hypothesized to act through numerous mechanisms, including biological, psychosocial, and community pathways (Hackett et al., 2016).

The social origins of mental health issues in First Nations contexts requires social solutions, and community‐based efforts are regarded as a key strategy to reclaiming and retaining holistic wellness among Indigenous peoples (Kirmayer, Simpson, & Cargo, 2003). Mental health literacy is one such approach that can help build individual and community resiliency. Mental health literacy arose as a concept in the 1990s when Anthony Jorm and others noted that in comparison to knowledge around promoting physical health, the public did not have the requisite knowledge and awareness to help prevent or respond to mental health challenges (Jorm, 2012). The concept of mental health literacy has been further developed into a multi‐faceted domain that includes understanding how to foster and maintain good mental health; understanding mental disorders and their treatments; decreasing stigma; and seeking help effectively (Kutcher, Wei, & Coniglio, 2016). In addition to low levels of mental health literacy, stigma creates an additional barrier to engaging in open and non‐judgmental conversations about mental health (Henderson, Evans‐Lacko, & Thornicroft, 2013). The Mental Health First Aid (MHFA) program was developed in Australia to address mental health literacy and stigmatizing attitudes (Kitchener & Jorm, 2002). MHFA applies a population health approach to mental health promotion by training people to recognize and respond to mental health problems and crises in others. The focus of the MHFA Basic course is learning a first aid action plan and specific skills.

A meta‐analysis of 15 published studies found that the MHFA Basic training program effectively increases participants’ mental health knowledge, reduces stigma, and increases behaviors that support individuals with mental health issues (Hadlaczky, Hökby, Mkrtchian, Carli, & Wasserman, 2014). MHFA Basic has been used internationally, but there have also been some attempts to create culturally relevant versions for specific populations. Cultural adaptation of the MHFA Basic training program was first undertaken in Australia for training among Aboriginal and Torres Strait Islander peoples. Findings from evaluations in Australia highlighted the importance of culturally appropriate and specific MHFA teaching materials (Hart et al., 2010). Although MHFA Basic has been used across Canada (including in First Nations communities), a similar need for adaptation was identified to make the program appropriate and relevant for First Nations contexts (Caza, 2010). Ensuring cultural relevance is critical, because other widely utilized mental health trainings used in First Nations contexts without adaptation have resulted in null or even negative impacts (Sareen et al., 2013).

In 2011, the Mental Health Commission of Canada (MHCC) set out to develop a version of MHFA that would be relevant for First Nations contexts. The approach taken was consistent with culturally adapted interventions, in that cultural activities and content remained embedded in Western theories of mental illness and health (Allen & Mohatt, 2014). The MHCC convened a guidance group of nationally recognized Indigenous leaders to advise them on this work. The fourth author of this paper served as a member of this group. Furthermore, MHCC hired an Indigenous consulting firm to assist in the early development of this work in conjunction with three First Nations partner sites. With respect to the partner sites, the structures of each site differed in that one was an urban Indigenous organization, while the others included an on‐reserve regional health authority, and a child and family services organization that provided services to fly‐in communities. Over a 3‐year period, many Indigenous individuals and organizations were involved with the development and piloting of the Mental Health First Aid First Nations (MHFAFN) course. In 2013, an additional three sites were added to the evaluation to inform further refinement of the course. Beginning in 2016, the MHCC began to offer MHFAFN more widely across Canada.

MHFAFN differs from MHFA Basic in a number of respects (MHCC, 2014); it situates mental health issues facing the current generation of Indigenous peoples as a social problem that stems from historical and contemporary forms of systemic colonial harms, and recognizes the need foster individual and community resiliency by building upon the natural healing resources embedded in First Nations cultures. MHFAFN has opportunities for community‐specific adaptations to ensure the content is relevant and respectful to the local Nation context, compared to the MHFA Basic which remains unchanged regardless of where it is offered. Three overarching components are woven throughout the course. First is the idea of *walking in two worlds*, or bringing together Western and Indigenous knowledge about mental well‐being. Second is a focus on *circles of support*—a community mapping exercise whereby participants identify available local supports and resources. Third, MHFAFN strategies are taught using the acronym EAGLE, which stands for: Engage and evaluation the risk of suicide or harm, Assist the person to seek professional help, Give reassurance and information, Listen without judgement, and Encourage self‐help strategies and gather community supports. In MHFA Basic, the acronym is ALGEE; however, this is not merely a re‐ordering of strategies, but an effort to adopt culturally meaningful symbols. Furthermore, the symbol highlights the importance of the eagle in many teachings and ceremonies as part of a vast social and spiritual network for many First Nations peoples.

The purpose of this study was to undertake a feasibility study of the MHFAFN to assess the acceptability of the intervention and cultural adaptation, and preliminary participant outcomes. A feasibility study is an appropriate framework for MHFAFN because it is a new adaptation of an existing program, and also because of the lack of effective programming for this context (Bowen et al., 2009). Specifically, we undertook a mixed methods evaluation to look at impacts on acceptability of the course (as reported by participants and facilitators), satisfaction with the cultural adaptation, and individual‐level impacts on knowledge, awareness, stigma, self‐efficacy and skills (Bowen et al., 2009). We approached this work from a perspectivism lens by enlisting stakeholders as co‐producers of knowledge, and explicitly addressing culture and contexts (Tebes, 2005; Tebes, Nghi, & Matlin, 2014).

## Methods

Our team embarked on this evaluation with a commitment to a two‐eyed seeing approach to data collection and interpretation; namely, wanting to bring together the strengths of Indigenous ways of knowing and Western ways of knowing (Iwama, Marshall, Marshall, & Bartlett, 2009). This stance paralleled the *walking in two worlds* approach of the MHFAFN course. Our overarching methodological framework embodied Indigenous community‐based participatory research (CBPR) principles, in that we valued contextual reflection, placed an emphasis on respectful and reciprocal research relationships that benefit communities, and prioritized Indigenous ways of knowledge transfer (Drawson, Toombs, & Mushquash, 2017).

We adhered to key ethical frameworks, including the OCAP (Ownership, Control, Access, and Possession) principles (First Nations Information Governance Centre, 2014) and the Tri‐Council Policy Statement: Ethical Conduct for Research Involving Humans (TCPS‐2; Canadian Institutes of Health Research, Natural Sciences and Engineering Research Council of Canada, and Social Sciences and Humanities Research Council of Canada, 2010). These two frameworks share several principles including the importance of research benefitting communities, access by members of the community to knowledge collected about them, research that is relevant to communities, and opportunities for co‐creation (Riddell, Salamanca, Pepler, Cardinal, & McIvor, 2017). We also adhered to a collaborative and community driven process throughout the evaluation by creating space for community members to co‐create relevant research questions, identify appropriate methods, and ensure data were being interpreted appropriately. While we proposed photovoice as a culturally relevant approach for data collection (Castleden, Garvin, & Huu‐ay‐aht First Nation, 2008), our community partners felt that the additional burden on participants and the time required for photovoice were more onerous than the benefits warranted, and directed us toward a strengths‐based survey and short face‐to‐face interviews instead.

With respect to our team, seven of the eight authors of this paper are Indigenous from diverse First Nations and Métis Nations across Canada. The Principal Investigator of the grant funding this work is the sole non‐Indigenous member of the research team. Recognizing that this could inadvertently create a power differential, we worked hard to ensure consensus‐based decision‐making by valuing all team member's voices and hearing all voices in a respectful way. We prioritized Indigenous knowledge to guide this work and ensure cultural relevance. Several of our team members work within an all‐Indigenous consulting organization that has undertaken program evaluation nationally and is often called upon to provide training or consultation for others doing similar work.

We used a mixed methods within an Indigenous CBPR approach, which is in alignment with Indigenous methodology frameworks (Drawson et al., 2017). Indigenous methodologies spans a range of approaches, but all of these share an emphasis on ensuring reciprocal benefits within the research relationship and co‐creating methodology. We were committed to these principles, but by virtue of having clearly defined objectives and methods at the outset of the evaluation (versus allowing these to emerge over the course of the project), we were not fully embodying an Indigenous methodologies approach. Mixed methods have the advantage of different data gathered from different approaches and sources being used to look at triangulation, complementarity, and enhancing significant findings (Andrew & Halcomb, 2007). We utilized quantitative data with caution, recognizing that researchers using positivist empirical research methods have significantly harmed Indigenous communities (FNIGC, 2014), but that there is also increased interest in reclaiming statistics as a means of conveying community stories to broader groups of stakeholders (Walter & Andersen, 2013). There is growing recognition that it is not the use of quantitative approaches that is in and of itself harmful, but rather that there is a need for reframing our approaches such that quantitative measures are used and data interpreted within a larger Indigenous methodology framework (Drawson et al., 2017; Roy, 2014).

In consultation with our community partners, we chose a retrospective pre‐post strategy, specifically a “post & then” design (Nimon, Zigarmi, & Allen, 2011, p. 12). Evaluators have noted that the retrospective pre‐post approach has the advantage of efficiency (i.e., requires only one administration), meets program developers’ and participants’ desire for face‐validity, and is particularly useful when a participant's frame of reference is likely to change over the course of an event, thus reducing shift bias (Hill & Betz, 2005). For example, in this study it might be difficult for participants to accurately rate their knowledge about the links between colonization and negative mental health at pre‐test, if they have not been exposed to those concepts before. Beyond these technical considerations, our team also identified ethical considerations involving pre‐post testing. Notably, many Indigenous individuals have had negative school experiences that may have resulted in negative connotations with written tests and reduced literacy. Beginning a course that is intended to bolster self‐efficacy and create safety with a “test” could potential trigger anxiety among those who had negative school experiences and/or result in a poor assessment of knowledge for those with reduced literacy. Furthermore, we viewed the retrospective pre‐post surveys as a more respectful approach in that it recognizes that individuals have the capacity to reflect meaningfully on their personal gains following an experience. However, we recognize that this approach has limitations, and that it is likely more effective at measuring subjective experiences of program‐related change (Hill & Betz, 2005).

### Participants

Participants attended one of ten MHFAFN course offerings across four provinces. Courses were offered in diverse locations, including First Nations communities, organizations in urban centres, and rural communities. Groups ranged in size from nine to 23 participants, with a total of 149 participants in the 10 groups. Seven of the 10 groups were comprised solely of Indigenous participants.

Overall, 91 participants completed the survey (mean age = 42.1 years, *SD* = 12.46; range = 19–73). It should be noted that missing data has resulted in different sample sizes for different analyses. Most participants self‐identified as being of Indigenous background (81.3% versus 15.4%), with three participants (3.3%) not answering the question. There was great diversity with respect to the Nations represented and how individuals self‐identified. Participants were given the option to identify their gender (versus being asked to check a box). All participants who answered this question identified as female (*n* = 70; 77%) or male (*n* = 19; 21%) and two participants did not answer (2%). Participants were also asked if they had previous mental health training. We coded responses for formal mental health training and identified three categories: (a) post‐secondary training (i.e., relevant diploma or degree courses regardless of whether the diploma or degree was completed); (b) relevant training course(s) such as ASIST, Mental Health First Aid Basic, therapeutic crisis intervention; and (c) no formal training. Because this was an open‐ended question, participants who left it blank were coded as having no formal experience (even if they did not write “none” in the answer box). The training groups were roughly evenly split, with 30.8% reporting relevant post‐secondary education, 38.5% reporting specific courses or qualifications, and the remaining 30.8% reporting no formal training. In addition, we conducted interviews with 89 participants during the latter half of the course. The timing on the interview was flexible to facilitate participants sharing their views at the time they felt most comfortable and ready, typically on breaks or at lunch on the final day of training. Although there were similar sample sizes for the surveys and interviews, there was only a 50% overlap (in terms of participants doing both surveys and interviews).

Nine of the 14 facilitators from the sites we visited participated in interviews. We also collected facilitator implementation surveys from 12 facilitators at site visits and an additional 12 at other courses (i.e., where MHFAFN was implemented but we did not do a site visit). The vast majority, of facilitators (but not all) were Indigenous. Ten of 14 facilitators we visited were female. All facilitators were working in the mental health field and in First Nations contexts. The questions in each tool had some overlap as well as unique areas to avoid inflation of findings.

## Measures

### Participant Interviews

Participants were asked five open‐ended questions relating to overall their experience of the course, participant outcomes, and the extent to which the course was perceived culturally relevant and safe. There was a range of interview durations (i.e., from 5 to 45 minutes).

### Participant Surveys

The participant survey was a 37‐item measure that was developed for this evaluation. There were six items addressing demographic variables (i.e., What cultural communities or Nations do you identify with?). There were five Likert‐type rating items assessing general satisfaction (e.g., satisfaction with the topics covered, satisfaction with the co‐facilitators), which required participants to rate items on a scale from 1 (*extremely dissatisfied*) to 5 (*extremely satisfied*). These five items were added to make an overall acceptability scale. There was space for additional comments following these ratings. Two items asked yes or no questions followed by space for comments (e.g., Do you think this course has helped increase your knowledge and understanding of cultural safety in Mental Health First Aid?), and two additional open‐ended questions asked about general learning and recommendations for changes to the course. The survey included 19 retrospective pre‐post Likert‐type items requiring individuals to rate their own knowledge, stigma, and self‐efficacy. There was also a scenario with three open‐ended questions. The survey is described in more detail below.

Knowledge and awareness, stigma, and self‐efficacy were all measured with Likert‐type retrospective pre‐post questions. There were two knowledge subscales, which required participants to rate their agreement with statements on a scale from 1 (*not at all*) to 5 (*very much*). The first measured knowledge related to mental health (six items). It included items such as “I am able to recognize emergency situations for self‐harm.” Despite the small sample size, these items showed adequate internal reliability (α = 0.89). The second knowledge subscale included items about the impacts of colonization, the role of culture in Indigenous mental health, and the importance of social support for well‐being. We labelled this subscale knowledge—social determinants of health (four items). This subscale included items such as “I know about the impacts of colonization on self, family, and community;” “I know about the link between culture and mental health;” and, “I am aware of the supports available to me as an MHFA responder.” The internal reliability for this subscale was adequate, especially considering the sample size and small number of items (α = 0.75). The stigma subscale (seven items) included items such as “I would be comfortable meeting a person with a mental health issue,” and “I would not be comfortable working with someone if I knew they had mental health problems.” Participants were required to rate their thoughts before and after training on a scale from 1 (*strongly disagree*) to 4 (*strongly agree*). Finally, there were two self‐efficacy questions,^2^ including “I am confident in my skills as a Mental Health First Aider,” and “I am able to listen without judgment.” For all subscales, a mean item score was calculated.

Skill application was explored with a scenario that participants were asked to respond to. The scenario described a situation where a friend was exhibiting potential signs of distress/depression and asked several follow up questions about how participants conceptualized what might be going on for this friend, what they would do, and how they would actually start a conversation.

### Facilitator Interviews and Surveys

Facilitator interviews included questions about perceived benefits of the course, feedback on the experience of delivering the training, and impacts (both expected and unexpected). The interviews were conversational (i.e., a relational process with a deep purpose of sharing a story as a measure; Kovach, 2010), included 16 questions and lasted between 25 and 105 minutes. The facilitator survey was conducted online. It consisted of 25 questions that asked about overall facilitation experience, any modifications made to the course, observed benefits among course participants, challenges in implementation, and experience with other MHFA courses.

### Procedure

Our research team conducted 10 site visits to observe the course and collect data from participants. Informed consent was obtained from all participants. All protocols were approved by the institutional research ethics board. We obtained research approval from each hosting organization and they sought appropriate community approvals. All interview and survey protocols are available from the first author.

Initially, the original procedure (which was co‐constructed with representatives from six sites) was for two of our researchers to attend the pilot site trainings, introduce the research, observe the course to make field notes, and conduct surveys at the end of the course. During our first three site visits, while we were adhering to the original procedure, the survey participation rate was very low (32%). After these first three site visits, our research team re‐grouped and decided to engage in a more relationship‐based evaluation approach consistent with Indigenous methodologies; instead of observing the courses, our team fully participated. As each course began, we introduced ourselves alongside course participants. In our introductions, we shared information about the research, explaining the importance of gathering feedback and how participants’ voices would be honored and reflected in the research. We remained engaged in the course throughout the duration of its delivery, taking field notes and intently listening to the participants when they expressed feedback over the two and a half days. We shared meals with participants in the evening, when invited. We also offered to interview the course participants when they wanted during course breaks or lunches, which allowed the participants to reflect on their experiences at the time of taking the course and provide oral feedback. This flexible interview framework led to more in‐depth answers in general than the paper survey was eliciting. After these changes were made, the survey participation rate increased dramatically and was 76% across the last six site visits. The interview participation rates for visits four through nine was 93%. Participants received a $10 gift card for participating in surveys, and an additional gift card of the same amount for their feedback through the interviews.

Facilitators were invited to participate in interviews near the end of the project, when most of them had had the opportunity to facilitate multiple courses. Facilitators from sites we visited were provided with an email link to complete an implementation survey following the course. The email link for the facilitator survey was also sent to additional facilitators that implemented the program but not at a site we visited. Facilitators who completed a survey received a $15 gift card for the survey and another for the interviews, reflective of the more numerous and in‐depth questions asked of facilitators than course participants.

We utilized member checking, which is an important Indigenous research method because it reinforces participants’ ownership and control over their own data (Drawson et al., 2017). After each site visit, we prioritized developing a community‐friendly two‐page summary of the findings from each site and provided it to the site within four weeks of the visit. In many cases facilitators had further email contact with the researchers on a range of matters related to the course.

### Data Analysis

Participant and co‐facilitator interviews and open‐ended survey questions were coded using an inductive approach to content analysis. These data were analyzed with a process of systematic coding by hand, and involved several rounds of open coding, grouping, and thematic categorization (Saldaña, 2015). A group coding procedure was used for these data, whereby the initial questions were coded by a small team of researchers, allowing for discussion around the essence of each data point and group consensus around the generation of emergent codes. Following this initial procedure, research team members coded individual portions of the data, with ongoing discussion around nuanced data.

Responses to the scenario questions were coded for content based on the presence of EAGLE strategies. A codebook was developed to distinguish among the strategies and provide examples. Participants were assigned a score based on one “point” for each example of an EAGLE strategy present in the scenario response. For example, a participant received a score of one if they provided a response with one example of the “Engage and evaluate the risk of suicide or harm” strategy. The total score maximum is five (when at least one strategy was provided for each of the EAGLE skills).

For the retrospective pre‐post data, we wanted to examine if individuals with different educational backgrounds rated themselves as having different starting points pre‐training, whether there was an overall increase from pre‐post, and whether there was an increase regardless of educational background. We analyzed the four subscales (i.e., knowledge‐mental health; knowledge‐social determinants of health; stigma; self‐efficacy) the same way. First, we conducted an ANOVA to evaluate whether there were differences in retrospective pre‐test ratings based on participants’ training background. Then, we used a paired samples *t*‐test to determine whether there was a significant difference in the retrospective pre‐post ratings for the whole sample. Next, we did a paired samples *t*‐test within each of the three training categories to see if there was an increase for each training background.

## Results

In this section, we present converging lines of evidence that highlight participants’ perceptions of the acceptability of the course generally, as well as more specifically related to the cultural adaptation. We then present gains in knowledge, awareness, and self‐efficacy, and reductions in stigma beliefs. We are intentionally privileging the voices of participants, and begin with data generated in participant and facilitator interviews before presenting the summaries of the retro pre‐post questions for each area of outcome analysis. Finally, we present participants’ responses to an open‐ended scenario.

### Acceptability of MHFAFN

Course participants in general were very accepting of the course and in interviews described it as much needed: “I get a sense of relief that finally there is a program like this for First Nations—it helps me think that it is addressing what we need to do in our communities.” (female participant, interview). Survey participants provided many general positive comments (23.9% of comments) including, “I was extremely satisfied, truly, with this course. It touched me in many ways ‐ healing ways.” (female participant, survey). Similarly, scores on the acceptability subscale analysis suggested a high level of satisfaction (*M *= 4.3, *SD *= 0.73).

### Satisfaction with the Cultural Adaptation of the Course

Overall, participants identified the inclusion of Indigenous‐specific content (i.e., historical content, the integrated Indigenous knowledge and culture) as a primary strength of the course. They further reflected that the MHFAFN course addresses the needs of First Nations. Similarly, many facilitators spoke about the value of including Indigenous‐specific components, including the historical content and cultural teachings, as well as incorporating fun and humor within the course. One facilitator shared, “I love that we train a course that has Anishinaabe content in it. It brings a different meaning and we can bring in our own experiences and perspectives to the course when we facilitate.” (female facilitator, survey).

Participants identified many aspects of the content of the MHFAFN course that worked well. Overall, it was shared that it was effective to walk in both worlds through integrating Indigenous knowledge and culture into the MHFA Basic course. Specifically, they mentioned that incorporating information about Canada's colonial history enhanced the cultural relevance and safety of the course. One participant shared:

It speaks well to the historical trauma, the shared trauma that Indigenous people have gone through. To help understand why people act in certain ways that aren't healthy for them. It will help to build a lot more empathy for when a person is going through something. We can say that these are signs of trauma and we can't just expect them to stop using their ways of coping overnight. (female participant, interview)

Ceremony, circles and prayer were also noted as important to include. Facilitators noted that including Indigenous‐specific content increased participants’ interest in the course. Additional aspects of Indigenous knowledge and worldviews that worked well included a focus on holistic health and looking at the whole person, learning about the medicine wheel, and having freedom to talk about culture and traditional teachings.

Despite the overall positive reaction of participants to the cultural content of the course, a minority of participants did raise concerns about the cultural components being underdeveloped or secondary to the western view of mental health. One participant who had already taken MHFA Basic found that the MHFAFN did not offer added value:

I found the content similar, it felt like a recertification, we took general then youth now this one, was looking for a little more FN content, teachings should have been more incorporated onto the content… I felt a little disappointed, I think too. It doesn't make it First Nations just the art and the feathers on it. I feel like there is not a lot of content except referring to a spiritual leaders in communities you should refer to, not content. (female participant, interview)

One participant noted concerns with how mental health diagnoses fit culturally: they gave the example of being diagnosed at a clinic with bipolar disorder, but from a cultural perspective, “When a person spirals spiritually it is them trying to bring in cultural reclamation.” While one participant thought there was too much spent time on the historical context, others recommended adding more information around historical trauma, and earlier colonial events.

Beyond wanting more cultural content, another participant noted that the need to stay on a schedule and meet specific curriculum objectives was not consistent with First Nations approaches to learning:

Maybe it is because they are rushing so much, they are not really interacting or letting people share. When you are First Nations you get to share when you need to. There is no time limit to sharing, that is something that bothers me. When they are talking and someone is sharing, they are not recognizing the sharing it just jumps back into the slide show… they are not putting our culture in it, I can't believe that they didn't mention the sacredness to some parts… Anyone could present and learn from the book or the slides but if it is not coming from the heart or if you are rushing then it is not culturally safe. (female participant, interview)

In terms of navigating between this tension of facilitating a First Nations learning experience and following the manual, one facilitator noted that s/he used the manual as a secondary source but taught from the heart.

It's there, I look at it, I read it, but it's natural for me. I just do it. It comes from my heart, history, how I live. I read it and see where we're at, but then I go on visions of my own, I know it's hard, I probably have a harder time because I have to put it in my Cree way of being taught, then put it in the Western way. That's how I've been taught, so that's how I do. I can bring in the story of what they're talking about. It's there for guidance. (female facilitator, interview)

### Participants’ Experiences of Increased Knowledge on Mental Health

Many participants identified the personal impacts that the course had for them as the most valuable aspects of the MHFAFN training. More specifically, participants identified an increase in knowledge and understanding as one of the most valuable personal impacts from the training (*n* = 27). Participants spoke about learning new mental health information and having increased knowledge of mental health, including the ability to recognize signs and symptoms and conceptualizing mental health as a continuum. For example, one participant referenced learning about the signs of suicidal ideation: “I really learned about risk factors/signs I wasn't aware of in terms of suicide ideation (e.g., giving away of things). Before, I never thought it was a sign, I would've accepted it as a gift, ‘thank you for thinking of me.’ That surprised me in terms of what I learned so far” (male participant, interview). Participants also spoke about having increased confidence in their knowledge and skills:

Because I don't have any counselling background, I never thought I was capable at helping people. I knew I could, but I didn't feel confident. But with this I think I would be able to help more in my job. I mostly work with youth programming. (female participant, interview)

Similarly, when facilitators were asked about the impacts of the training for participants, they most commonly identified areas of increased knowledge and awareness (*n* = 6).

For the retrospective pre‐post rating scale results, an ANOVA showed a significant difference across training groups in self‐reported knowledge about mental health at pre‐test *F*(2, 88) = 11.41, *p* = 0.000. Post hoc analysis showed that participants with courses or post‐secondary work rated themselves higher than those with no formal training, but there was no difference between those with courses and post‐secondary. There was a significant increase in self‐reported knowledge about mental health from pre‐test (*M *= 2.90*, SD = *0.70*)* to post‐test (*M *= 3.60*, SD = *0.43), *t* (87) = −11.50 (*p* = .000). When pre‐post differences were examined for each training background group separately, each showed a significant increase in self‐reported knowledge.

### Gains in Culturally Relevant Knowledge and Social Determinants of Health

Participants noted that they learned new information with respect to history, culture and traditions. One participant remarked on the importance of this culturally relevant knowledge for non‐Indigenous peoples:

There's a lot of people that don't know our traditions. From a perspective of not living in community, this would be effective as a teaching component for non‐Indigenous people and how that historical experiences that we have had can impact our trauma and the mental health issues that we experience. (female participant, interview)

Similarly, facilitators identified important outcomes related to increased understanding of Indigenous teachings in the training. This was particularly poignant, they noted, for non‐Indigenous staff: “It's like fireworks, they get a better understanding of who we are as First Nations people and who we come from. [It is] culturally sensitive. They get to realize what it is like to be the two‐eyed seeing” (female facilitator, interview).

Both facilitators and participants identified gaining an increased awareness of community supports via the Circles of Support activity, in which participants identify available community resources. “Seeing it up on the wall like that, there is so much help out there for people, we just need the guidance to find that support, this course will help us share that this knowledge is out there” (female participant, interview).

For retrospective pre‐post ratings on the knowledge about social determinants of health, there was an overall significant difference at pre‐test across training‐level groups *F*(2, 88) = 4.87, *p* = 0.01, but the only significant difference was between those with post‐secondary training and those with no formal training (see Table 1). Similarly, for knowledge about social determinants of health, there was a significant increase in self‐reported knowledge from pre‐test to post‐test for the whole sample (*t*(88) = −9.26, *p* = 0.000) (see Table 2).

**Table 1:** Differences across training groups at pre‐test

Scale	Postsecondary *M* (*SD*)	Other training/certification *M* (*SD*)	No formal training *M* (*SD*)	*F* (*df*)
Knowledge‐MH	3.23 (0.62)^a^	3.04 (0.62)^a^	2.47 (0.63)^b^	11.41 (2, 88)***
Knowledge‐SDOH	3.39 (0.51)^a^	3.19 (0.63)^a,b^	2.78 (0.67)^b^	5.37 (2, 88)***
Stigma	2.08 (0.58)^a^	1.94 (0.42)^b^	2.28 (0.70)^b^	7.47 (2, 88)***
Self‐efficacy	3.19 (0.79)^a^	3.16 (0.63)^a^	2.46 (0.64)^b^	10.52 (2, 88)***

a and b denote equivalent or different means at the *p* < 0.01 level.

***p < 0.001

**Table 2:** Retrospective pre‐ and post‐test ratings on knowledge, and self‐efficacy for whole sample and by training group

Scale	Sample size	Pre‐test *M* (*SD*)	Post‐test *M* (*SD*)	*t* (*df*)
Knowledge‐MH (all)	*n* = 88	2.90 (0.70)	3.60 (0.43)	−11.50 (87)***
Post‐secondary	26	3.25 (0.64)	3.77 (0.31)	−5.22 (25)***
Other training	34	3.03 (0.63)	3.69 (0.38)	−7.14 (33)***
No training	28	2.47 (0.63)	3.40 (0.50)	−8.10 (27)***
Knowledge—SDOH (all)	*n* = 89	3.12 (0.65)	3.67 (0.38)	−9.26 (88)***
Post‐secondary	26	3.40 (0.52)	3.78 (0.29)	−3.89 (25)***
Other training	35	3.19 (0.63)	3.71 (0.37)	−5.38 (34)***
No training	28	2.78 (0.66)	3.51 (0.43)	−7.04 (27)***
Stigma (all)	*N* = 90			
Post‐secondary	25	2.08 (0.58)	1.89 (0.56)	3.01 (25)*
Other training	36	1.94 (0.43)	1.74 (0.32)	4.10 (35)**
No training	29	2.28 (0.54)	2.14 (0.64)	ns
Self‐efficacy^a^ (all)	*n* = 91	2.94 (0.76)	3.67 (0.43)	−11.08 (88)***
Post‐secondary	26	3.17 (0.81)	3.77 (0.35)	−4.30 (25)**
Other training	35	3.16 (0.63)	3.77 (0.39)	−6.69 (34)***
No training	28	2.46 (0.64)	3.45 (0.46)	−9.41 (27)***

^a^Stigma is scored such that higher scores reflect higher stigma beliefs.

*p < .05. **p < .01. ***p < .001.

### Participant Changes in Stigma Beliefs

Participants discussed stigma in their interview in terms of personal impact and also the importance of public education more broadly. One male interview participant reflected directly on his attitude shift, “My understanding of some of the issues that people have lived through or have to live with, my attitudes have softened.” Another interview participant reflected more generally on the role of education in breaking down stigma, “The information about the mental health illnesses and diagnoses, because a lot of our community members don't know about them, so I think that's important which will break down the stigma within our communities and within our people.” (female participant, interview)

Retrospective pre‐post ratings on stigma beliefs demonstrated an overall significant difference at pre‐test across training‐level groups *F*(2, 87) = 3.60, *p* = 0.05; interestingly, the post‐secondary and no formal training groups did not differ from each other, but the other training group reported lower stigma beliefs (see Table 1). Overall there was a self‐reported decrease in stigma beliefs from pre‐test to post‐test, but because subgroup analysis showed that the no formal training group did not report a significant decrease, we only report the pre‐post scores by group (see Table 2).

### Participant Gains in Self‐Efficacy and Skill Development

Participants spoke about understanding the importance of the First Aider skill of active listening and non‐judgmental conversation; this skill includes being mindful of body language, eye contact, and tone when talking to someone in crisis. Similarly, during our participation throughout the site visits, we heard from participants that they felt more equipped to support people in distress by applying EAGLE actions:

Right now they are telling us about EAGLE – I didn't know about that, that will be helpful because it guides us what to say, how to react, to keep calm and keep the person calm and not be judgmental (female participant, interview)

Facilitators commonly identified participant outcomes related to confidence and new skills, noting that participants are gaining self‐confidence in their employment and are better able to recognize people in need of support. One facilitator said:

I think it has given a lot more self‐confidence to the workers to the various degrees in being more flexible in dealing with the clients … they have conveyed that they feel better at being able to do their jobs more effectively and their sense of validation for being a care giver has been raised, has been validated, it improves confidence levels (male facilitator, interview)

Facilitators made similar observations on their implementation surveys about course participants’ gains in self‐efficacy, “They stated they were more confident in helping people in a crisis” (female facilitator, survey).

As with knowledge questions, there was a significant difference in pre‐intervention self‐efficacy associated with the different categories of background training (*F*(2, 88) = 7.47, *p* = .001). Tukey's post hoc tests showed that the difference was between the two groups with formal training (post‐secondary or other courses) and the group with no formal training (see Table 2). Finally, there was a significant increase from pre‐test ratings of self‐efficacy (*M *= 2.94*, SD = *0.76) to post‐test (*M *= 3.67, *SD *= 0.43), *t*(88) = −11.08, *p* = .000.

### Impacts Beyond Skills, Attitudes, and Self‐efficacy

Participants and facilitators identified aspects of the course that surprised them and shared personal impacts. Both course participants and facilitators identified self‐reflection as an important outcome for participants. Participants noted that the course allowed them to reflect on their personal experiences, as well as the ways they can apply the teachings to their work. Both participants and facilitators noted that the participants were making both personal and historical connection with the course content.

When I was a younger, after coming out of residential school, I wondered why I drank, did drugs, it wasn't until later I realized the issues, residential school was never a topic in mainstream schools and the conditions and the impacts… this kind of training helps me understand the parts and the roots… the roots were cut off and the tree died when we were taken away, now we have to replant the seed so we grow re‐vibrant again, that's what we're doing with this course – we're replanting; this course is one of those new roots, language revitalization is another root, culture is another root, then on the top we have the tree where we want to be. (female participant, interview)

Throughout the course the participants began to share very personal and powerful experiences. One participant was able to talk openly about a family tragedy for the first time and thanked us for allowing him the opportunity to gain some understanding of the tragic events. Many participants indicated that they grew in knowledge and in spirit throughout the course. (female facilitator, survey)

Participants spoke about learning from other participants in the room and the positive impacts of sharing stories (*n* = 5), including learning new strategies and traditional teachings from each other. They also noted the ways in which MHFAFN has contributed to their own healing journeys (*n* = 4); for example, some participants noted that the group discussions provided important opportunities to talk about historical trauma, while others identified an increased desire to find balance and holistic wellness: “What I've gotten from a day and a bit of this course is that it is important to sit and re‐evaluate and try to find balance” (female participant, interview). For one participant, their participation in a sharing circle through MHFAFN represented the first time that they felt safe to share in a group:

The residential school part triggered some stuff in me that I didn't think would. But it's a good thing. It's never happened to me before, I was avoiding it I guess. That's the first time I've said in a circle that I've been to residential school. (female participant, interview)

### Application of Mental Health First Aider Skills and Strategies

In response to the scenario about a hypothetical friend who is described as suffering from a possible mental health crises, all respondents successfully identified that they were concerned about John and that he might be dealing with an issue that required assistance (100% of respondents). Even though the questions did not prompt participants to use EAGLE strategies, participants described approximately three EAGLE strategies in their responses (*M* = 2.96, *SD = *1.31). Overall, the most popular EAGLE strategy participants described they would use in their scenario responses was “Engage and evaluate the risk of suicide or harm,” with 79.1% of all participants identifying this technique. Females tended to use more EAGLE skills (*M = *3.20, *SD = *1.17) than males (*M = *2.37, *SD* = 1.30) in their responses (*t* (*df* = 87) = 2.68, *p* = .009). When the individual strategies were examined with Pearson's chi‐square analyses, we found that females used the “Engage” strategy at significantly higher rates than males did. Females also used the “Give reassurance and encourage self‐help strategies” more than males at a difference that approached trend significance (i.e., *p* < 0.1). Sample responses, frequencies, and gender differences are provided in Table 3.

**Table 3:** Use of EAGLE strategies in response to mental health scenario by gender

	Examples	Overall (%)	Females (%)	Males (%)	χ^2^
E Engage and Evaluate the risk of suicide or harm	“I would ask John if I could speak with him and explain that I have noticed the change in his behavior and I am very concerned about him. I would ask him how he is doing and why he is feeling the way he is feeling, I would ask him if he has had thoughts about suicide because of how he is feeling…”	79.1	82.9	68.4	χ^2^(1) = 1.93, *p* = 0.14
A Assist the person to seek professional help	“… ask if he would accept help from a mental health resource and help him find one he feels comfortable going to.”	46.2	52.9	26.3	χ^2^(1) = 4.22, *p* = 0.04
G Give reassurance and information	“That he is not alone (connect to resources, be available to talk). That there is help available. That in time, things will pass.”	51.7	57.1	36.8	χ^2^(1) = 2.47, *p* = 0.09
L Listen without judgement	“…listen, be present, and validate.”	60.4	62.9	57.9	χ^2^(1) = 0.16, *p* = 0.44
E Encourage self‐help strategies and gather community supports	“…I would attempt to support John in a good way (if he is First Nations) possibly connecting him with Elders, Mental health councilors, Offering care support being encouraging and caring letting him know about other resources… I would acknowledge his gifts and see if I could build upon that.”	58.2	64.3	42.1	χ^2^(1) = 3.05, *p* = 0.07

## Discussion

This article presents the findings of a mixed methods feasibility evaluation of the acceptability and preliminary outcomes of the Mental Health First Aid First Nations course. One strength of this study was the diversity across the groups included. Some trainings were offered within organizations for their own staff (e.g., a health authority, friendship centre), while other groups used open registration and resulted in highly diverse groups with respect to age, professional roles, and previous training. Furthermore, across groups, participants described a range of motivations for attending; these included personal and professional motivations, as well as being directed to attend by a supervisor. We utilized course participants’ self‐reports of acceptability of the course, gains in knowledge and self‐efficacy, changes in stigma beliefs, answers to a vignette, and interview responses, as well as facilitator interview and implementation survey data.

Overall participants reported a high level of acceptability; however, when they were asked more pointed questions about cultural relevance some participants offered more specific critiques of the program. Perspectivism provided an important framework in this regard, by acknowledging the role of context and culture. It also highlighted the variability in participants’ experience, most notably that although most participants viewed the cultural content as a strength, some felt the cultural context was inadequate. Participants described gains in knowledge related to mental health signs and symptoms, but also to a broader contextual understanding of well‐being. In addition, they reported increased self‐efficacy and decreased stigma beliefs. There was a high degree of convergence across data sources and methods, increasing our confidence in our findings. Participants’ voices shared through interviews gave context and depth to the gains documented in quantitative pre‐post ratings.

### Limitations

The findings reported in this article should be considered within the context of our study's methodological limitations. First, our overall sample size did not facilitate many subgroup analyses. In particular, the small number of male participants makes any findings regarding gender differences very tentative. Second, although we highlighted the advantages of the retrospective pre‐post approach in our methods section, this approach also has well recognized limitations. Participants may feel a desire to show a learning effect and make the workshop presenters look effective. In addition, recall of information is imperfect and may create a threat to validity. An empirical comparison of learning measured with retrospective pre‐post and objective pre‐ and post‐measures of knowledge gains following an educational program found that retrospective pre‐test was an accurate way of *identifying* learning, but not as accurate at *quantifying* it (Bhanji, Gottesman, de Grace, Steinert, & Winer, 2012). Thus, this evaluation was likely more successful in identifying the presence of learning rather than quantifying the change. However, it should also be noted that some of the types of learning that participants described are not easily measured with an objective test of facts (i.e., the contextualization of mental health within a colonial history and the personalization of information). Another possible limitation is that although emphasizing relationships with our participants increased the cultural safety and authenticity of the research process, it could also potentially create a hesitation to openly criticize the course in an in‐person context. We sought to minimize this potential bias by offering multiple avenues for providing feedback (i.e., opportunities for critiques via the confidential survey). Finally, having pre‐set objectives as a feasibility evaluation may have limited our ability to identify other important themes present in the qualitative data.

### Individual and Community Impacts of MHFAFN

The significant variability in participants and groups gave us the opportunity to look at whether the course was more effective for some individuals than others. Interestingly, although the course was not designed for a professional audience, participants reported benefits across knowledge and self‐efficacy, regardless of their previous training. Thus, MHFAFN is an attractive public health approach because it offers benefits to a wide range of participants, regardless of their gender, previous experience and training.

Participants demonstrated good application of the MHFAFN material in response to a hypothetical scenario about an acquaintance exhibiting signs of a potential mental health crisis. Participants readily applied the EAGLE strategies and were able to identify actual conversation openers. Although female participants utilized a higher overall number of EAGLE strategies and more use of some specific strategies, both male and female participants were able to identify practical and effective strategies for responding.

The results of this evaluation suggest that MHFAFN is a feasible, acceptable, and potentially effective approach for promoting mental health literacy in First Nations contexts. The extent to which the cultural adaptation struck the right balance for participants varied; while all participants found the added cultural context to be valuable, some felt that the adaptation did not go far enough and still privileged western concepts of mental health and illness. Participants’ increases in knowledge and skills were consistent with meta‐analytic findings of MHFA Basic in general (Hadlaczky et al., 2014); however, participants in our study described a positive impact that went beyond learning the signs and symptoms of mental health crises and how to respond to them. Their interview responses conveyed a deeper personal impact that took different forms for different participants. For some it was the first venue where they could feel safe sharing their own struggles; for others, there was an epiphany about the links between residential schools and current mental health struggles. The course helped participants link the historical and systemic contributors to poor health outcomes among First Nations people today. The results of this feasibility study provide an important foundation for further evaluation and more rigorous study of the effectiveness of MHFAFN.

## Conflicts of Interest

This research was funded by a Canadian Institutes of Health Research (Population Health Intervention Research) grant to Claire Crooks. Kim van der Woerd was a member of the original guidance group put together by the Mental Health Commission of Canada to guide the development of the Mental Health First Aid First Nations program. Kim van der Woerd is the principal member of Reciprocal Consulting, an all‐Indigenous research and consulting firm that has played a number of roles in the development and evaluation of the MHFAFN. Monique Auger, Billi Joe Rogers, Sam Tsuruda, and Cassidy Caron are all paid employees of Reciprocal Consulting. This firm provided some consultation on the development of the MHFAFN program. They also were contracted to assist with the evaluation (by C. Crooks). In some ways this could constitute a conflict of interest, but at the same time, deep relationships and involvement over the course of the project provide a strong foundation for research in Indigenous contexts.

## Ethical Compliance

We complied with all ethical principles put forth by APA. Furthermore, we obtained ethical approval from our institutional Research Ethics Board. Because this work was conducted with Indigenous individuals and communities, we endeavored to meet additional ethical considerations, as described in the body of the paper.

## Notes

1 First Nations include the first inhabitants of Canada. They are one of the three most commonly recognized Indigenous groups in Canada: First Nations, Inuit and Métis. While the term First Nations is used in Canada, the term Native American is used in the United States.

2 We did not calculate a reliability for a self‐efficacy subscale because there was only two questions.
